# An Arrhythmia Classification Model Based on a CNN-LSTM-SE Algorithm

**DOI:** 10.3390/s24196306

**Published:** 2024-09-29

**Authors:** Ao Sun, Wei Hong, Juan Li, Jiandong Mao

**Affiliations:** 1 School of Electrical and Information Engineering, North Minzu University, North Wenchang Road, Yinchuan 750021, China; 20227378@stu.edu.cn (A.S.); 20217372@stu.edu.cn (W.H.); 2 Key Laboratory of Atmospheric Environment Remote Sensing of Ningxia, North Wenchang Road, Yinchuan 750021, China

**Keywords:** arrhythmia, CNN-LSTM-SE, classification prediction

## Abstract

Arrhythmia is the main cause of sudden cardiac death, and ECG signal analysis is a common method for the noninvasive diagnosis of arrhythmia. In this paper, we propose an arrhythmia classification model based on the combination of a channel attention mechanism (SE module), convolutional neural network (CNN), and long short-term memory neural network (LSTM). The data of this model use the MIT-BIH arrhythmia database, and after noise reduction of raw ECG data by the EEMD denoising algorithm, a CNN-LSTM is used to learn features from the data, and the fusion channel attention mechanism is used to adjust the weight of the feature map. The CNN-LSTM-SE model is compared with the LSTM, CNN-LSTM, and LSTM-attention models, and the models are evaluated using Precision, Recall, and F1-Score. The classification performance of the tested CNN-LSTM-SE classification prediction model is better, with a classification accuracy of 98.5%, a classification precision rate of more than 97% for each label, a recall rate of more than 98%, and an F1-score of more than 0.98. It meets the requirements of arrhythmia classification prediction and has a certain practical value.

## 1. Introduction

An arrhythmia is a condition in which the heart rhythm is abnormal or irregular [1]. It can manifest as a fast, slow, erratic heartbeat and in severe cases can lead to a heart attack or sudden death. There are several arrhythmias, including sinus arrhythmia, ventricular arrhythmia, atrial arrhythmia, atrial fibrillation, and ventricular fibrillation. The classification of arrhythmias is complex; some malignant ventricular arrhythmias can trigger sudden cardiac death within minutes. Due to the sudden and random nature of arrhythmias, it is difficult for the vast majority of patients to receive professional cardiopulmonary resuscitation promptly, resulting in an out-of-hospital resuscitation success rate of less than 1 percent in China, making it a major health risk for the population. The best way to reduce disease morbidity and mortality and improve the health of the elderly is to achieve early prevention and early diagnosis of disease.

With an increase in age, the number of apoptotic cardiomyocytes in the cardiovascular system will increase, the structure and function of conduction tissues will change, and the prevalence of cardiovascular diseases will also increase. A large number of reports show that the incidence and mortality of cardiovascular diseases in China are highest in the world. The clinical diagnosis of arrhythmia is based on the characteristics of the electrocardiogram (ECG). Therefore, doctors are required to have a strong professional ability and rich experience, but there are problems such as a long discrimination time and low efficiency. Consequently, an increasing amount of research has commenced to investigate the application of machine learning methods for classifying arrhythmias [2].

Machine learning algorithms can acquire knowledge from extensive datasets and autonomously classify and categorize arrhythmias. Some commonly employed machine learning algorithms include support vector machines, Random Forest, and Deep Neural Networks. These algorithms can automatically extract the specific features of ECG signals, such as the amplitude of the R-wave [3], QRS waveform width, and P wave shape, and classify arrhythmias by analyzing the training datasets.

Deep learning-based arrhythmia classification algorithms have been extensively researched and utilized in recent years [4]. For instance, techniques utilizing convolutional neural networks (CNN) can automatically extract the spatial and temporal characteristics of ECG signals [5], thereby improving the classification accuracy of arrhythmias. Additionally, methods based on recurrent neural networks (RNN) can model the time series of ECG signals to better deal with complex arrhythmias [6].

As early as 2014, Yan et al. proposed an ECG beat classification algorithm based on cluster analysis, which takes advantage of the characteristics of the small intra-individual variability of ECG beats, adopted a two-stage cluster analysis, sampled representative ECG beats, and combined with the auxiliary diagnosis of the ECG physician to achieve the accurate classification of ECG beats [7]. In 2016, Wang et al. proposed a novel approach for extracting features from ECG signals by combining empirical mode decomposition (EMD) with approximate entropy [8]. In 2020, Huang et al. proposed a method aimed at achieving the precise and intelligent classification of arrhythmias by utilizing a fast compression residual CCN as an accurate classification method for an intelligent ECG classifier [9]. In 2021, Mohebbanaaz introduced an enhanced K-nearest neighbor classifier to categorize arrhythmia beats. Empirical findings demonstrate that the suggested technique can enhance the classification precision of the KNN classifier when confronted with extensive datasets [10]. In 2022, Mohammed M. Farag presented a lightweight, self-contained CNN model for classifying ECG signals and detecting arrhythmias in real time using the Short-time Fourier Transform. The results achieved by the proposed classifier enable it to be deployed on a wide range of arrhythmia-monitoring edge devices [11]. In the same year, Sowmya et al. proposed a classification method for ECG signals and arrhythmia signals based on the CNN and long-term memory neural network (LSTM) deep learning model. Through comparison, it was found that the CNN-LSTM model has better dataset validation [12]. In 2023, Li et al. presented a novel ECG classification technique utilizing a Convolutional Vision Transformer and multimodal picture fusion. The experiment shows that the classification method can effectively improve the diagnostic efficiency of cardiac diseases such as arrhythmia and myocardial infarction [13]. In the same year, Nahak et al. proposed a new method for classifying 17 classes of arrhythmias using wavelet scattering transform [14]. In 2024, Zhang et al. suggested Multi-scale Convolutional Transformer Networks (MCTnet) as an effective fusion of transformer encoders to combine CNNs to classify ECG signals. The results demonstrate that MCTnet can surpass existing deep learning models, underscoring its efficacy in classifying ECG signals [15].

The existing methods used to study ECG signals are usually based on the R-R interval, which needs to determine the position of the beat. Most studies use the position of the R-peak as a reference point to divide the beat. There are two ways to determine the position of the R-peak: one is based on the annotation of the R-peak by experts in the database, and the other is calculated by the existing R-peak detection algorithm. For example, the Pan-Tompkins algorithm can achieve 99.3% accuracy in locating the beat [16]. However, because some arrhythmic heartbeats have very close peak values, the algorithms used to identify these heartbeats could introduce errors. This results in discrepancies between the obtained data and the real data, producing different heartbeat shapes when performing segmentation on the same data. And because of the calibration of heartbeats by experts, it is difficult to ensure that all heartbeats can be marked quickly and without omissions. Therefore, in actual research, the above two methods have their disadvantages. Deep learning is prominent in pattern recognition applications, which can adaptively and automatically learn complex and representative features directly from data [6], reducing over-reliance on manual feature extraction.

For some classic models, traditional deep neural network (DNN) models perform poorly in handling the temporal dependencies of ECG data. A conventional CNN has fixed feature channel weight distributions, lacking adaptive adjustment capabilities. While standalone LSTM models outperform CNNs in spatial feature extraction, they are inadequate for handling complex ECG signals alone. Although CNN-LSTM combines both advantages, it still shows insufficient precision in feature channel weight distribution and lacks targeted optimization.

To address the aforementioned issues, this study has put forth a classification model for arrhythmia, which combines the channel attention mechanism (SE module), CNN, and LSTM. The data of this model use the MIT-BIH arrhythmia database, the EEMD denoising algorithm was used to denoise the original ECG data, a CNN-LSTM was used to learn the features of the data, and the channel attention mechanism was fused to adjust the weight of the feature map. Compared to other classic models, the CNN-LSTM-SE model overcomes the limitations of single models in processing ECG signals by integrating the spatial feature extraction capabilities of CNN with the temporal processing power of an LSTM and further enhances model performance through the adaptive adjustment of feature channel importance by the SE module. Some experiments were used to verify the proposed method. The results show that the CNN-LSTM-SE classification prediction model has excellent classification performance, meets the requirements of arrhythmia classification prediction, and has a certain practical value.

## 2. Common Arrhythmia Types

Arrhythmia is a disease triggered by an abnormal source of electrical stimulation of the heart or an abnormal process of electrical signal conduction. By analyzing the characteristics of electrocardiogram (ECG) signals, many types of arrhythmias can be effectively diagnosed. This paper is based on the MIT-BIH arrhythmia database, which contains data on many types of arrhythmias. We selected four common types of arrhythmia and normal rhythm data according to a mechanism of arrhythmia occurrence for classification and prediction analysis [17]. The common types are as follows:

(1) Atrial premature beat

**Figure 1:**
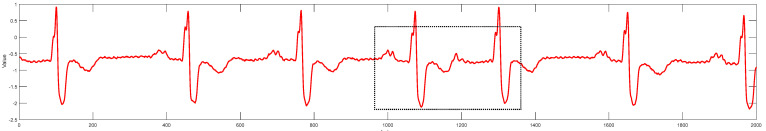
An atrial premature beat electrocardiogram from record 118 in the MIT-BIH dataset.

An atrial premature beat (APB) is an early impulse from an ectopic pacing site in the atria that leads to the early depolarization of the atria, thus triggering early atrial contraction [18]. Its main electrocardiographic features include the following: the presence of an early atrial P’ wave with a morphology that is markedly different from that of the sinus P wave in the same lead; the coupled intervals are usually fixed, and if they are not, the possibility of multiple atrial premature beats of multiple origins or parallel rhythms should be considered; atrial P’ waves are often accompanied by supraventricular QRS-T waves, with P’R intervals of greater than 120 milliseconds; and the compensatory intervals are usually incomplete [19]. The ECG of atrial premature beats in the MIT-BIH dataset is shown in Figure 1, with the dashed frame highlighting the typical characteristics of this category.

(2) Premature ventricular contraction

**Figure 2:**
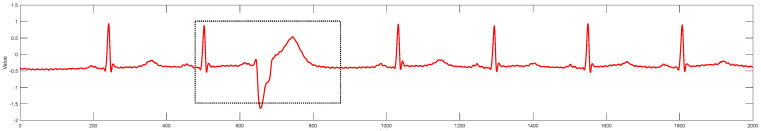
A ventricular premature beat electrocardiogram from record 205 in the MIT-BIH dataset.

Premature ventricular contractions are abnormal beats that occur when an ectopic pacing site within the ventricle sends an impulse ahead of time instead of being initiated by the normal sinus node or atrioventricular node, resulting in premature contraction of the ventricle [20]. Key features of premature ventricular beats on the ECG include the following: a cluster of wide and sharp QRS waves that appear early, are 120 milliseconds or longer in duration, and are not preceded by a P wave; the interassociative interval is fixed in most instances, that is, if more than one premature ventricular beat occurs on the same ECG, the difference in the interassociative interval is usually less than 80 milliseconds; and when the sinus rhythm is regular, the compensatory intervals are usually complete [21]. In addition, there are secondary changes in the ST-T wave, with the direction of the T wave in premature ventricular beats being opposite to the direction of the main wave of the QRS wave cluster, and changes in the ST segment. The ECGs of premature ventricular beats in the MIT-BIH dataset are shown in Figure 2.

(3) Left bundle branch block

**Figure 3:**
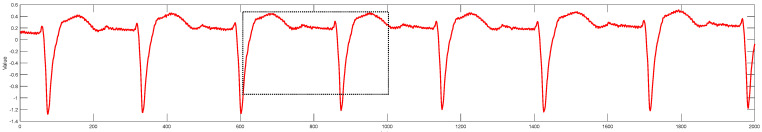
A left bundle branch block electrocardiogram from record 109 in the MIT-BIH dataset.

The left bundle branch block (LBBB) refers to a blockage or delay in the conduction of the left bundle branch. Under normal conditions, electrical impulses originate from the sinoatrial node, pass sequentially through the atrioventricular node, the His bundle, the left and right bundle branches, and finally reach the ventricles, causing ventricular depolarization and contraction. However, in the case of LBBB, the electrical signal cannot pass through the left bundle branch and instead first travels through the right bundle branch to the right ventricle, then spreads to the left ventricle via myocardial fibers, altering the sequence of ventricular depolarization. This abnormal conduction pathway leads to characteristic changes in the electrocardiogram [22]. The main features include the following: a QRS complex typically exceeding 120 milliseconds due to the delayed depolarization of the left ventricle, resulting in a widened QRS complex; in lead V1, the QRS complex usually appears as a deep and broad S wave, with the absence of a normal R-wave, forming a “W” -shaped pattern. Additionally, due to abnormal depolarization, secondary changes in the ST segment and T wave occur, with their direction opposite to the main QRS wave [23]. The ECG of the left bundle branch block in the MIT-BIH dataset is shown in Figure 3.

(4) Right bundle branch block

**Figure 4:**
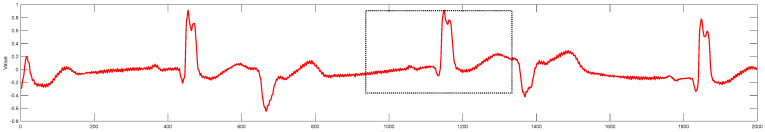
A right bundle branch block electrocardiogram from record 207 in the MIT-BIH dataset.

The right bundle branch block (RBBB) refers to a conduction delay or blockage in the right bundle branch, preventing the electrical signal from being transmitted normally through the right bundle branch. Instead, the signal first passes through the left bundle branch to the left ventricle and then through the ventricular muscle fibers to the right ventricle. Similar to the LBBB, the RBBB results in a prolonged QRS complex, typically exceeding 120 milliseconds, due to the delayed depolarization of the right ventricle [24]. Key electrocardiographic features include the “rsR” pattern in leads V1 and V2, characterized by a small initial R-wave followed by a wide and delayed R-wave. This morphology is often described as “rabbit ears” or an “M shape” in the right-sided leads [25]. The electrocardiogram of the RBBB in the MIT-BIH dataset is shown in Figure 4.

## 3. Methodology

### 3.1. CNN Algorithm

**Figure 5:**
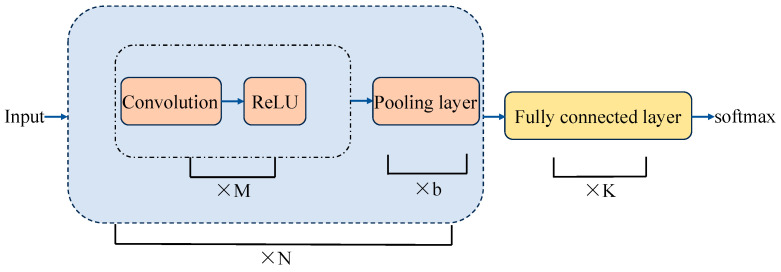
CNN structure.

CNN is one of the most effective deep learning models for solving various application problems [26]. A CNN model typically consists of four different types of layers: convolutional layers, activation layers, pooling layers, and fully connected layers. The overall structure of a commonly used CNN is shown in Figure 5.

In the task of arrhythmia classification prediction, the CNN model utilizes 1D convolutional layers to process the time-series data of ECG signals. Initially, the input signal undergoes convolution, which is similar to feature extraction in the spatial domain, where the signal is convolved with multiple filters to capture features across different frequency bands. These convolutional layers perform pointwise multiplication with the input data to extract local patterns in the signal, such as sudden waveform changes. The convolutional layer is calculated as follows:

(1) $$y_{j}^{\left(l\right)}=f(\sum_{i\in M_{j}}w_{ij}^{l}*x_{i}^{\left(l-1\right)}+b_{j}^{\left(l\right)})$$

where $y_{j}^{\left(l\right)}$ is the output of the first convolution layer, f denotes the activation function, M is the input feature map, ω is the weight value of the convolution kernel, $x_{i}^{\left(l-1\right)}$ is the input of the first layer, and b is the bias [27].

ECG data contain many local features, such as P waves, QRS wave clusters, and T waves. Convolutional layers use kernels to scan the ECG signals, capturing these local features, removing noise from the ECG signals, and enhancing useful signal characteristics. Through multiple layers of convolution, the model can gradually filter out irrelevant noise and highlight the key features of the ECG signals.

Additionally, convolutional layers apply different filters along the time axis to capture patterns with temporal dependencies in the ECG signals. For example, the morphology and duration of the QRS complex are crucial indicators for diagnosing heart diseases, and convolutional layers can effectively extract this information. In this way, convolutional layers not only improve the model’s ability to recognize ECG signals but also enhance its robustness and accuracy when processing time-series data.

Next, the model applies pooling layers to compress the dimensions of the feature maps, which helps reduce computational complexity while retaining the essential features. The pooling layers select the maximum value within local regions of the feature map, further enhancing the model’s robustness and selectivity for important features.

Finally, the model combines the extracted features through fully connected layers and ultimately classifies the input into different categories of arrhythmia. Through this multi-layered structure, the CNN effectively captures temporal patterns and local features in the ECG signals, leading to accurate arrhythmia classification.

### 3.2. LSTM Algorithm

The LSTM network is a model improved from the RNN, specifically designed to handle and transmit information across long sequences of data [28]. Its main advantage lies in effectively avoiding the issues of vanishing and exploding gradients commonly seen in a traditional RNN, ensuring that important information from long ago is not lost [29].

**Figure 6:**
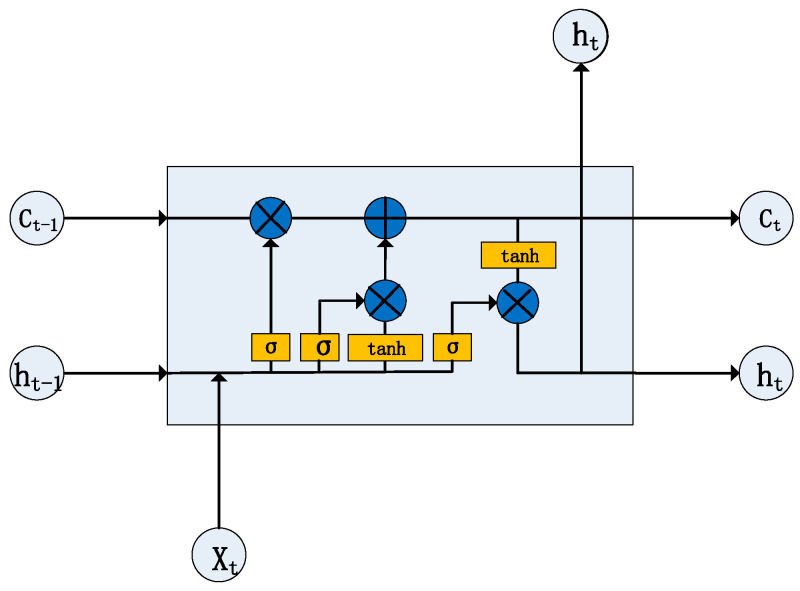
The structure diagram of the LSTM unit network.

At the core of the LSTM are three gates (input gate, forget gate, and output gate) and a memory cell. By controlling these gates, the LSTM can dynamically adjust the content of its memory based on current inputs and past information, allowing it to retain key features when processing time-series data. This structure makes the LSTM particularly well-suited for handling complex data with temporal dependencies, such as ECG signals. The structure of the LSTM unit network is shown in Figure 6.

The role of the input gate is to extract the valid information in the input vector by the tanh function and then filter and control the input of this information by combining it with the sigmoid function. The computation formula is given by

(2) $${\tilde{C}}_{t}=\tanh W_{c}\cdot \left[h_{t-1},x_{t}\right]+b_{c}$$

(3) $$i_{t}=\sigma \left(W_{i}\cdot \left[h_{t-1},x_{t}\right]+b_{i}\right)$$

where $W_{i}$ and $W_{c}$ denote the weight matrix, and $b_{i}\;$and $b_{c}$ are bias terms [30].

The forget gate works by deciding which memories need to be forgotten based on the new input received and the output from the previous moment. Specifically, the input data receive $f_{t}$ after passing through σ. If $f_{t}$ is 0, the data will be discarded; if $f_{t}$ is close to 1, the data will be retained. The formula for calculating the forget gate is written as in [31]:

(4) $$f_{t}=\sigma \left(W_{f}\cdot \left[h_{t-1},x_{t}\right]+b_{f}\right)$$

where σ is the sigmoid function, $W_{f}$ is the weight matrix, $\left[h_{t-1},x_{t}\right]$ represents merging two vectors into a vector of greater length, and $b_{f}$ is the bias term.

The role of the output gate is to extract the information from the vectors of the current input and the output of the previous moment through the sigmoid layer, which is processed by the tanh function, and then the dot product of the two can be used to obtain the output of the LSTM at the moment *t* [32]. The output gate is the vector of the current input and the output of the previous moment. The formula for the above process is given by

(5) $$O_{t}=\sigma (W_{o}\cdot \left[h_{t-1},x_{t}\right]+b_{o})$$

(6) $$h_{t}=O_{t}\cdot tanh(C_{t})$$

where $W_{o}$ is the weight, and $b_{o}$ is the offset term.

### 3.3. Channel Attention Mechanism

Channel attention is a technique that aims to demonstrate the relationship between different channels. It automatically determines the significance of each feature channel through network learning and assigns weight coefficients to each channel. This process enhances important features and suppresses non-important features. SENet pioneered the channel attention mechanism; its core is the Squeeze and Excitation (SE) module, which is used to collect global information, capture channel relationships, and improve feature representation capabilities [33].

**Figure 7:**
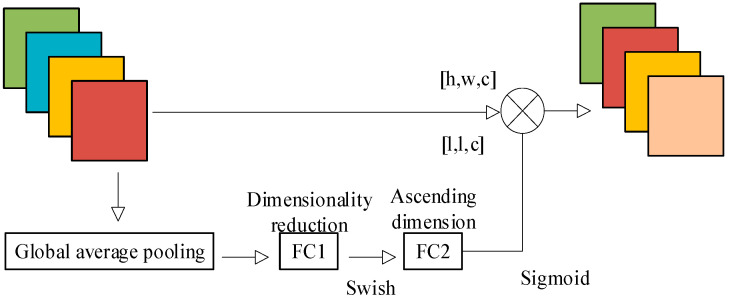
SE module realization flow.

During the Squeeze phase, the SE module utilizes the global averaging pooling operation to compress the two-dimensional characteristics of each channel into a single real number. This compression reduces the feature map from a size of [*h*,*w*,*c*] to [1,1,*c*] [34]. The process of the SE module is shown in Figure 7.

During the Excitation stage, the full connection layer (FC1) is utilized to decrease the channel dimension of the feature map vector to its original size of 1/*r*, specifically [1,1,*c* × 1/*r*]. Next, the Swish activation function is applied. After that, the feature map vector is sent through a fully connected layer (FC2) to restore its dimensions to [1,1,*c*], that is, the original size. Then, it is converted to a normalized weight vector between 0 and 1 by the sigmoid function.

The Scale operation performs element-wise multiplication between the normalized weights and the channels of the input feature map, resulting in the creation of a weighted feature map.

### 3.4. CNN-LSTM Arrhythmia Classification Model Based on SE Module

This model optimizes its parameters by combining CNN, LSTM, and SE modules and experimenting with multiple learning rates and batch sizes, to improve accuracy and model performance for ECG signal classification [35].

This model utilizes a CNN as its primary architecture, with an SE module added to extract effective features and suppress less important ones. The preprocessed data are fed into the model, where it is first processed by three convolutional and pooling layers. The first layer, Conv1D, uses 128 filters with a convolutional kernel size of 20 and a stride of 3 and is equipped with batch normalization and max-pooling layers. The second layer, Conv1D, uses 32 filters with a convolutional kernel size of 7 and a stride of 1 and is also equipped with batch normalization and a max-pooling layer. The third layer, Conv1D, uses 32 filters with a convolutional kernel size of 10 and a stride of 1 and is used in conjunction with the SE module to dynamically adjust the weights of the features [36]. After these convolutional and pooling operations, the data are passed through an LSTM layer to extract temporal features. The LSTM layer is followed by a flattening layer to unfold the 3D feature map into a 1D vector. Subsequently, a Dropout layer is used to prevent overfitting, and the results are passed through two fully connected layers (using 20 and 10 neurons, respectively, with ReLU as the activation function). Finally, the 5-class classification results are produced through the Softmax activation function. The detailed layer structure information of the model, including input dimensions, output dimensions, kernel sizes, pooling sizes and other related information, is listed in Table 1.

**Figure 8:**
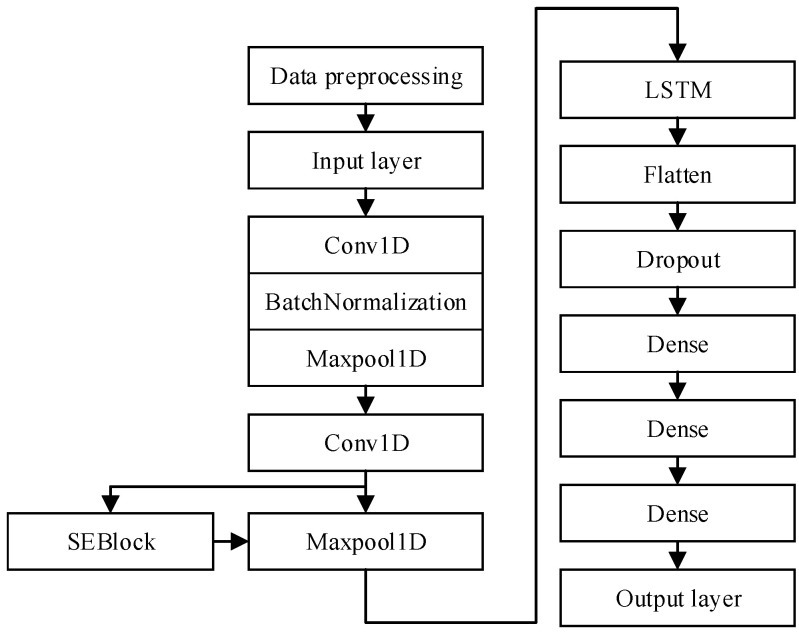
The structure block diagram of the CNN-LSTM-SE model.

In the model optimization stage, several learning rates and batch sizes were set for experiments; the training, validation losses, and accuracy rates under each parameter were recorded; and the loss rate and accuracy rate curves were plotted. Finally, the classification performance of the model was evaluated by calculating the confusion matrix and F1 score, and the best parameters were selected for final training. The structural block diagram of the model is shown in Figure 8 [35].

With the deepening of the training degree, the neural network model will appear with an overfitting problem. Therefore, we generally increase the number of dataset and sample diversity, add Dropout regularization, and add an Early Stopping mechanism. To suppress overfitting as much as possible, the Dropout layer and Batch Normalization layer are added to the network [37], and the Adaptive Moment Estimation (Adam) optimizer is used to optimize the model parameters [38].

## 4. Data Preprocessing

**Figure 9:**
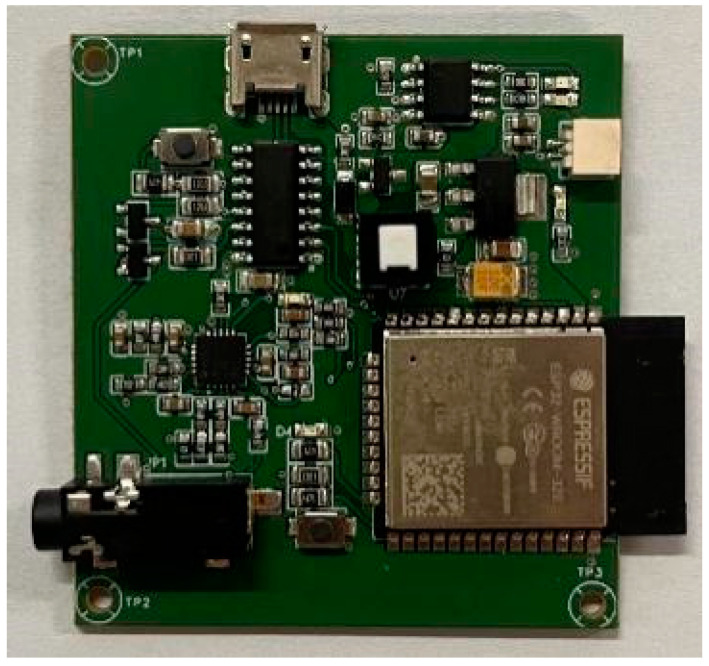
The portable single-lead ECG monitor developed independently.

This ECG signal data used to model analysis come from the MLII lead in the MIT-BIH dataset. Before entering the model, the data undergoes some preprocesses, including filtering for noise reduction, Z-score normalization, R-wave detection, and ECG segmentation. Additionally, the real ECG signal data containing baseline drift, collected using a single-lead ECG monitor developed by the Ningxia Hui Autonomous Region Key Laboratory of Atmospheric Environment Remote Sensing, was used to further validate the effectiveness of the filtering algorithm when applied to real-world ECG signals. Figure 9 shows the portable single-lead ECG monitor independently developed.

The system first collects the original ECG signals of the human body through patch electrodes and sends them to the front-end acquisition circuit. After hardware filtering and amplification processing, the processed signal is transmitted to the A/D sampling channel of the microcontroller unit (MCU) for signal conversion. The obtained digital signal is filtered through a digital filter designed by MCU programming and sent to the low-power Bluetooth (BLE) of the MCU. Finally, it is sent to the mobile terminal through BLE. In this system, the ESP32 is used as the MCU chip, and the ECG signal is measured by the AD8232 sensor [35].

### 4.1. EEMD Decomposition Algorithm

The fundamental concept behind the EEMD algorithm is to utilize the statistical properties of white noise with a uniform frequency distribution. By repeatedly adding Gaussian white noise to the signal, the signal will exhibit continuity across different scales and alter the characteristics of its extreme points. This promotes anti-mixing decomposition, effectively preventing mode aliasing and end effects. The algorithm consists of the following individual steps:

(1) Add normal distributed white noise $N_{i}(t)$ of equal length to the original signal X(t) several times, that is, where

(7) $$Y_{i}\left(t\right)=X\left(t\right)+N_{i}(t)$$

where $Y_{i}(t)$ is the signal after adding white noise for the *i*th time [39].

(2) The Intrinsic Mode Function (IMF) component $C_{ij}(t)$ and margin $r_{i}(t)$ are obtained by EEMD decomposition of $Y_{i}(t)$, where $C_{ij}(t)$ represents the j component obtained by decomposition after adding white noise for the *i*th time [40].

(3) The overall average of each component $C_{ij}(t)$ is calculated to counteract the influence of numerous additions of white noise on the real IMF, based on the assumption that the statistical mean of uncorrelated random sequences is zero. The EEMD decomposition finding is presented as follows:

(8) $$C_{j}=\frac{1}{n}\sum_{i=1}^{n}C_{ij}\left(t\right)$$

where n is the number of times white noise is added.

We used the eemd.eemd() function from the PyEMD library to perform EEMD decomposition on the signal, extracting all IMFs. Since the first IMF often contains the trend component, we removed it using u_eemd = u_eemd[1:, :]. During the Fourier transform, the fft function from scipy.fft was applied to perform the Fast Fourier Transform. Given the symmetry of the spectrum, we plotted only the first half of the spectrum to simplify the visualization and highlight the main frequency components.

### 4.2. Noise Reduction Effect Evaluation Index

The metrics commonly used to evaluate denoising performance include the correlation coefficient, signal–to–noise ratio (SNR), and root mean square error (RMSE). The correlation coefficient is a measure of the relationship between two variables, and it can be used to assess the similarity between IMF components and the original signal. The greater the number of correlations, the higher the similarity between the IMF component and the original signal [41]. SNR is used to evaluate the relative strength of the signal to noise. A higher SNR indicates less noise in the signal, suggesting better denoising performance. RMSE measures the difference between the denoised signal and the original clean signal. A smaller RMSE value indicates that the denoised signal is closer to the original signal, implying better denoising effectiveness. The formulas for the correlation coefficient, SNR, and RMSE are defined as follows:

(9) $$R\left(IMF_{i},y\right)=\frac{cov\left(IMF_{i},y\right)}{\sqrt{cov\left(y\right)}\sqrt{cov\left(IMF_{i}\right)}}\;cov\left(IMF_{i},y\right)=\sum_{i=1}^{N}(IMF_{i}-\bar{IMF})(y-\bar{y})\;cov\left(IMF_{i}\right)=\sum_{i=1}^{N}{\left(IMF_{i}-IMF\right)}^{2}\;cov\left(y\right)=\sum_{i=1}^{N}(y-\bar{y}{)}^{2}$$

(10) $$SNR=10lg\{\frac{\sum x(t{)}^{2}}{\sum [x\left(t\right)-x(t{)}^{\prime }{]}^{2}}\}$$

(11) $$RMSE=\sqrt{\sum [x\left(t\right)-x(t{)}^{\prime }{]}^{2}/n}$$

where y is the mixed signal combined with the original signal and noise, $\bar{y}$ is the average value of the original signal and the mixed signal, x(t) represents the value of signal x at time t, $x(t{)}^{\prime }$ represents the value of the denoised signal at time t, and n represents the number of signal samples [42].

### 4.3. Filter Denoising Processing

**Figure 10:**
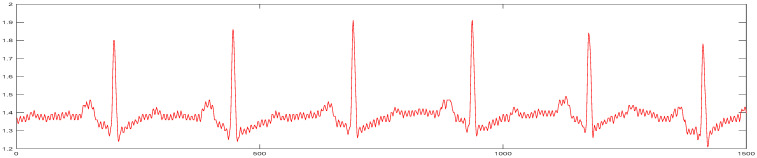
Actual measured original ECG signal.

In this paper, the actual measured signal is used to verify the filtering and denoising effect. The actual measured ECG signal is shown in Figure 10.

**Figure 11:**
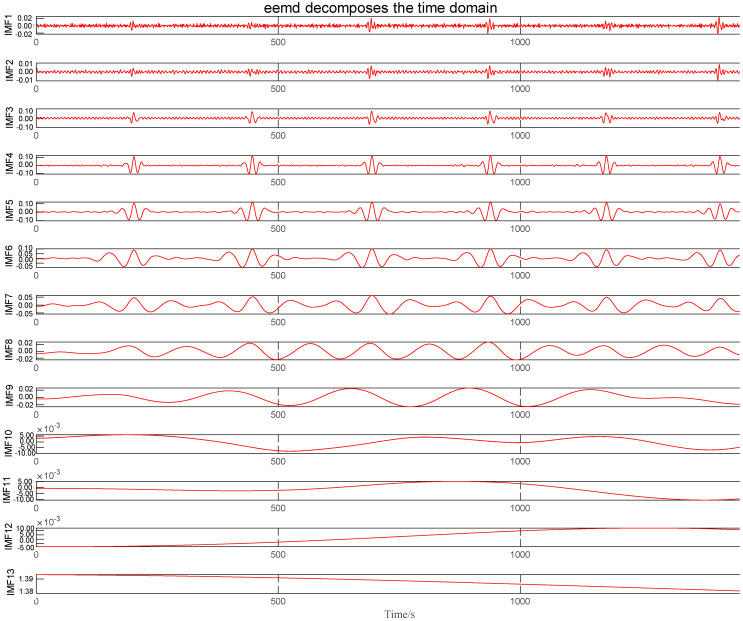
IMFs after EEMD decomposition.

There is a lot of interference in the process of ECG measurement. After the signal is decomposed by the EEMD method, the inherent mode diagram is obtained. Figure 11 shows the IMFs after EEMD decomposition.

**Figure 12:**
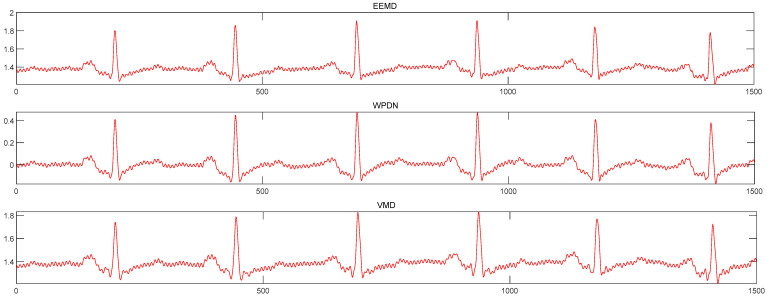
The denoising effect of the three methods.

To make a comparison, this paper compares the EEMD method with the wavelet packet denoising method and VMD denoising method, and calculates the SNR, correlation coefficient, and RMSE of the three methods. Figure 12 shows the denoising effect of the three methods.

Table 2 lists the evaluation index of the noise reduction effect of the three methods. It can be seen that the EEMD denoising method has the highest SNR, the smallest RMSE, and the best correlation coefficient compared with the other two methods. Since the experimental signal is the same and other conditions remain unchanged, the method proposed in this paper achieves the highest SNR compared to the control method, indicating that the mode-mixing problem has affected the denoising performance. Meanwhile, the wavelet packet denoising, VMD denoising, and EEMD denoising methods maintain good amplitudes, and the waveforms after denoising are smooth. Only certain points exhibit oscillation, but the EEMD method is comparatively gentler than the other two methods, which indicates that the method proposed in this paper is superior to the contrast noise reduction method in addressing the mode aliasing problem. It is capable of preserving the valuable information of the original signal while efficiently eliminating noise.

### 4.4. R-Wave Localization and ECG Segmentation

**Figure 13:**
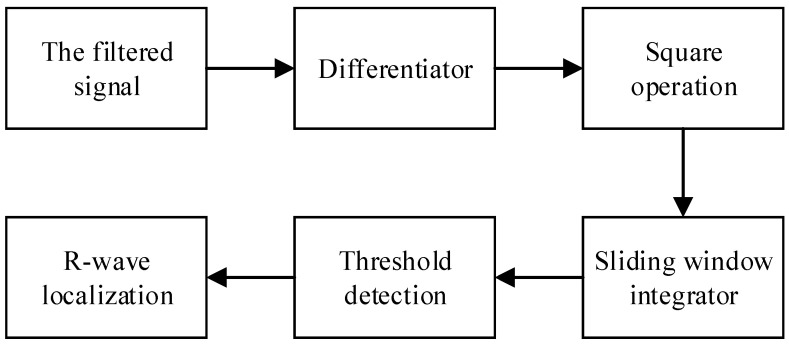
R-wave positioning process.

The R-wave is the most obvious peak of the QRS complex waveform and contains the most useful information of features of the whole heartbeat. In this paper, the Pan–Tompkins algorithm is adopted for R-wave positioning. Figure 13 shows the flow chart of the R-wave positioning process.

**Figure 14:**
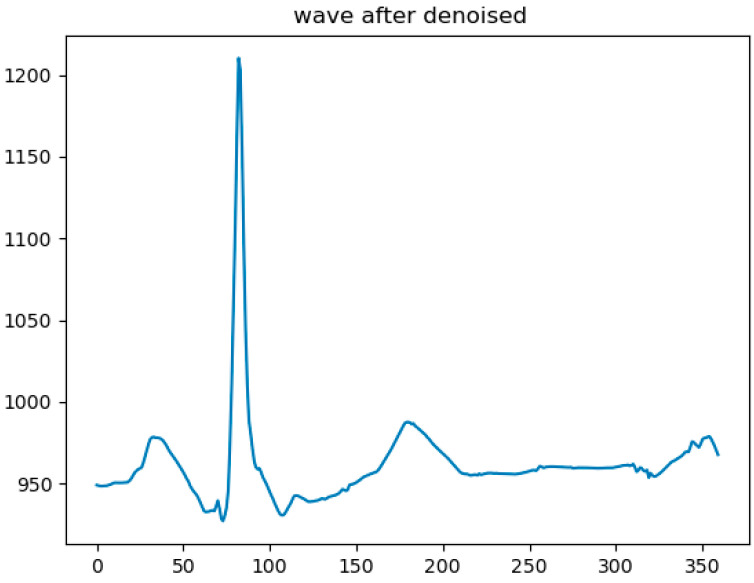
The visualization of the input after ECG segmentation.

The input of the model requires a fixed-length ECG signal fragment, so it is necessary to intercept the ECG fragment using the position of the R-wave peak as the input signal. Since the hardware sampling frequency in this paper is 360 Hz, 179 points are selected forward, 180 points are selected backwards, and a total of 360 points are selected together with the R-wave peak; one second of the ECG fragment data are used as an input. The average heartbeat of a person is usually once in 0.8 s, so the ECG signal selected for one second can contain two R-wave peaks or a complete heartbeat so that more useful features can be obtained for the model to extract. The visualization of the input after ECG segmentation is shown in Figure 14.

### 4.5. Z-Score Standardization

Z-score standardization helps to extract and classify features in ECG signals. By standardizing the signal data, it is possible to highlight and amplify the characteristics of the signal, such as R-wave peaks and patterns. Moreover, ECG signals usually have different amplitude ranges and units. Z-score standardization can convert the amplitude of the ECG signal into a standard deviation relative to the entire signal set, standardizing it into data with similar scales, and helping to eliminate the effect of amplitude differences between different signals. The calculation formula of Z-score standardization is given by

(12) $$Z=\frac{x-\mu }{\sigma }$$

where *Z* is the standardized output value of the Z-score, μ represents the average value of sample data, and σ is the standard deviation of sample data.

We used the stats.zscore function from the SciPy library to perform Z-score normalization on the signal, ensuring it has a mean of 0 and a standard deviation of 1. This process helps eliminate amplitude differences between different ECG signals, ensuring the data are processed on a uniform scale, thereby improving the performance and accuracy of the classification model.

### 4.6. Data Balancing

When processing the MIT-BIH ECG database, data balancing is an indispensable step. In an imbalanced dataset, the number of samples in minority classes is significantly lower than in majority classes, which may cause the classifier to lean towards predicting the majority class, thus reducing the classification accuracy for the minority class. Additionally, in such imbalanced datasets, the model may overfit the majority class and ignore the minority class. By balancing the data, the model can achieve more balanced classification performance across all categories, thereby improving its generalization ability.

In this paper, both oversampling and under-sampling methods are used for data balancing. Oversampling involves generating new samples in the feature space to increase the number of minority class samples, or simply randomly duplicating minority class samples to increase their quantity. Undersampling involves randomly removing samples from the majority class to reduce their quantity.

**Figure 15:**
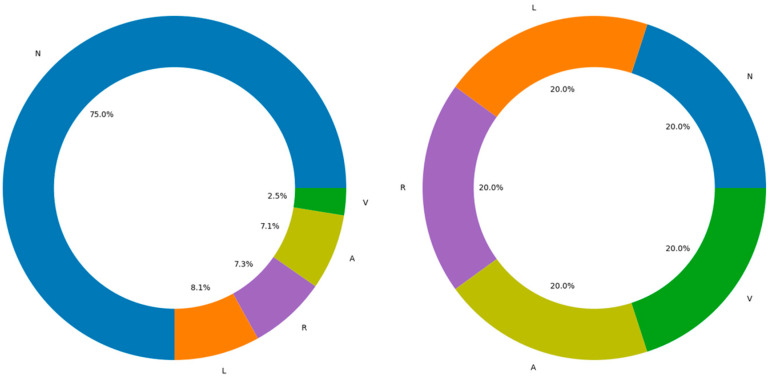
MIT-BIH ECG database data before and after balance distribution comparison.

In the dataset, the number of samples in class N (75,011 groups) is far greater than in the other classes (A, V, L, R), which have 8071, 7255, 7129, and 2546 groups, respectively. This imbalance may cause the classifier to favor predicting class N, leading to the neglect or misclassification of minority classes. After applying oversampling and undersampling techniques, the sample size for each class (N, A, V, L, R) has been balanced to 5000 groups, resulting in a total of 25,000 groups. The distribution of the MIT-BIH ECG database before and after data balancing is shown in Figure 15.

### 4.7. Dataset Partitioning and Model Validation

After data balancing, to prevent overfitting and optimize model parameter settings, we further split a portion of the training data to serve as a validation set. At the end of each training epoch, the model is evaluated on the validation set to monitor the learning progress and adjust model parameters. We used the train_test_split function to randomly divide the dataset into training and testing sets. Of the total dataset, 70% is used for training the model, while the remaining 30% is reserved for final performance evaluation. The test data do not participate in the training process; instead, they are used to evaluate the model’s performance and assess their generalization ability.

### 4.8. Feature Extraction and Processing

During the signal processing and feature extraction process, a CNN-LSTM-SE model was constructed using TensorFlow and Keras frameworks. The model automatically extracts the spatial features of ECG signals through one-dimensional convolutional layers (Conv1D) and optimizes the feature maps with channel weighting using the SE module, enhancing important features while suppressing irrelevant information. The model then captures the temporal dependencies of the signals through the LSTM layer, improving the classification ability for complex arrhythmias. After feature extraction and optimization, the refined signal features are passed through fully connected layers and classified using the Softmax activation function. By combining the spatial feature extraction of the CNN, the temporal modeling capability of the LSTM, and the channel optimization mechanism of the SE module, this model effectively enhances the accuracy of arrhythmia classification. Additionally, learning rates and batch sizes were fine-tuned during the implementation to ensure optimal performance.

## 5. Model Training and Performance Analysis

**Figure 16:**
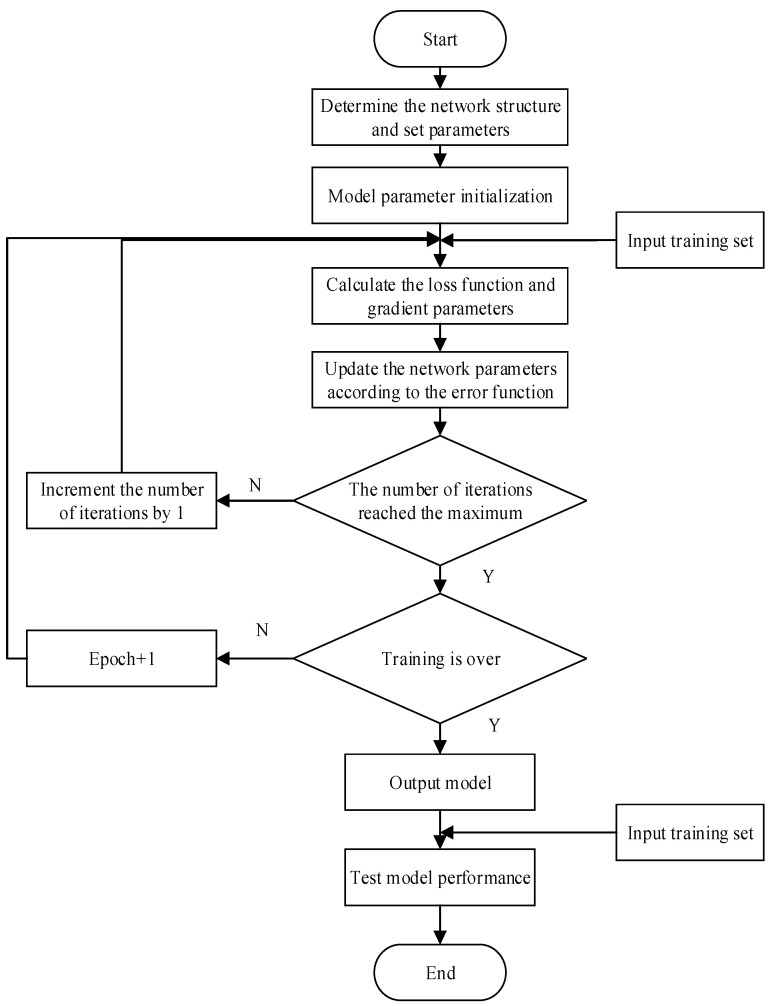
The training flow chart of the CNN-LSTM-SE model.

Figure 16 illustrates the training flowchart of the CNN-LSTM-SE model. In this design, two key parameters are considered in particular: learning rate and batch size [35]. To achieve optimal performance in arrhythmia classification prediction, parameter optimization is an essential step. To evaluate the significance of learning rate and batch size in the proposed model, we conducted multiple comparative experiments with different parameter sets.

**Figure 17:**
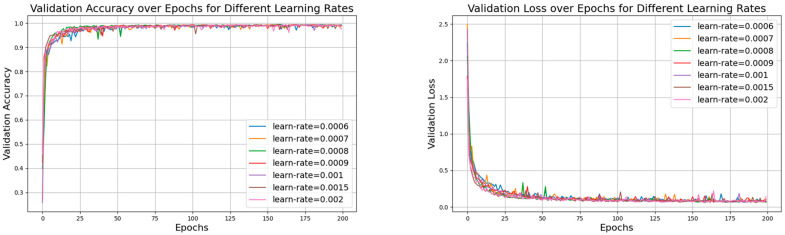
The validation accuracy and loss for different learning rates with a batch size of 150.

**Figure 18:**
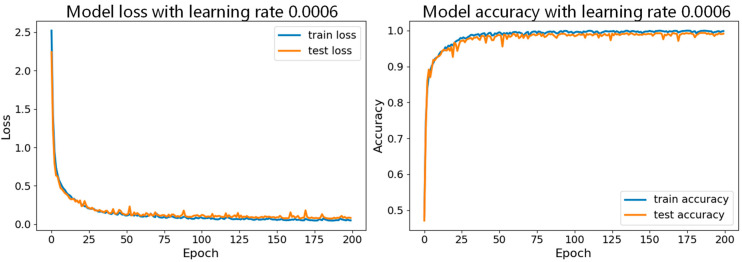
Comparison of model performance for learning rates of 0.006 with batch size of 150.

**Figure 19:**
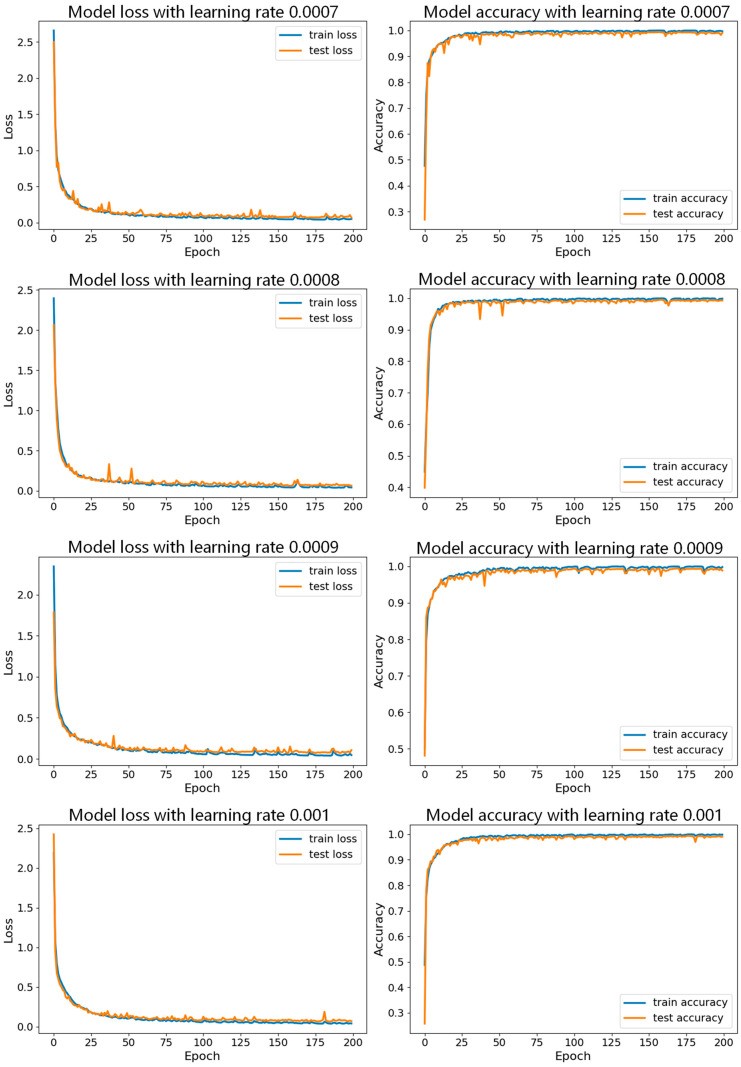
Comparison of model performance for learning rates of 0.0007, 0.0008, 0.0009, and 0.001 with batch size of 150.

**Figure 20:**
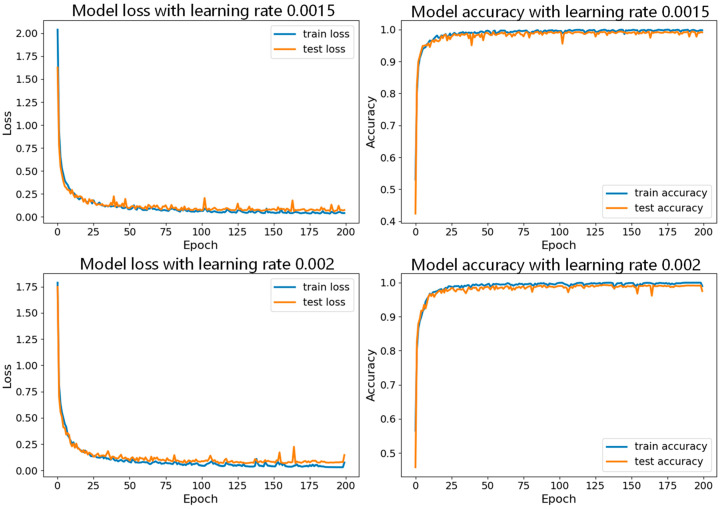
Comparison of model performance for learning rates of 0.0015 and 0.002 with batch size of 150.

First, while keeping the batch size constant, we tested the model with different learning rates, with specific parameter sets shown in Table 3. In these experiments, we set the number of iterations to 200. Figure 17 shows the accuracy curves for the seven datasets with a batch size of 150, and Figure 18, Figure 19 and Figure 20 show a comparison of the model performance at different learning rates.

From Figure 17, the curve for the learning rate of 0.001 is relatively smooth with minimal fluctuations, indicating that the model performed more stably throughout the training process, consistently maintaining high accuracy and effectively avoiding issues of overfitting or underfitting. In Figure 19, the curve for the learning rate of 0.001 drops rapidly during the initial stages of training and then remains at a low level in subsequent stages. In contrast, the curves for the other six learning rates show significant fluctuations in validation loss, which could lead to large errors during training and potentially cause overfitting. Therefore, compared to the other learning rates, the learning rate of 0.001 demonstrates superior stability and reliability.

**Figure 21:**
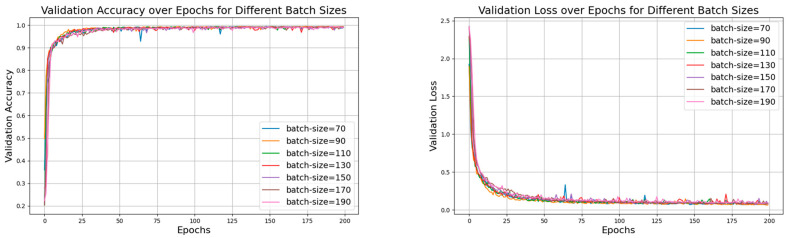
Validation accuracy and loss for different batch sizes with learning rate of 0.001.

**Figure 22:**
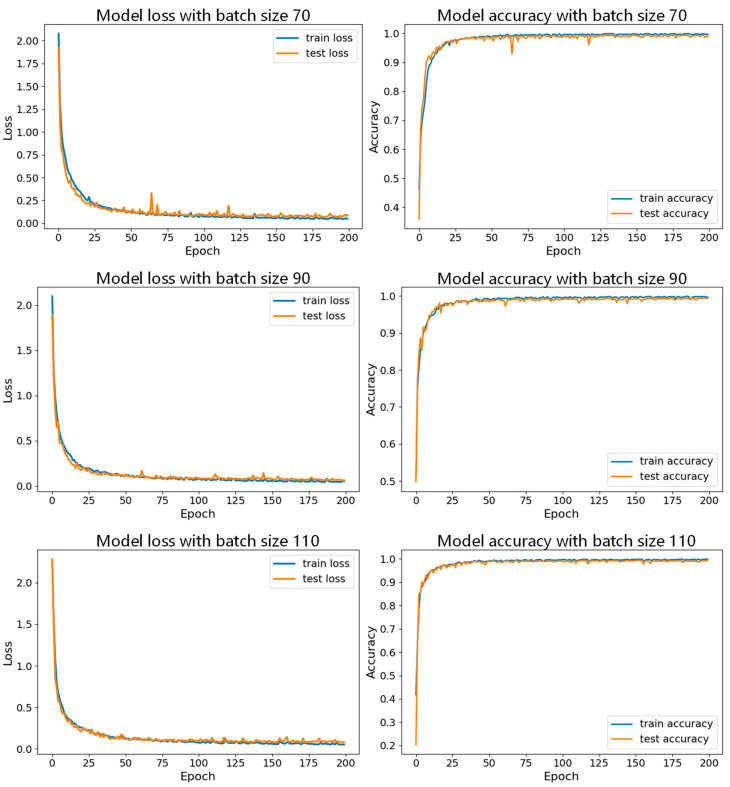
Comparison of model performance for batch sizes of 70, 90, and 110 with learning rate of 0.001.

**Figure 23:**
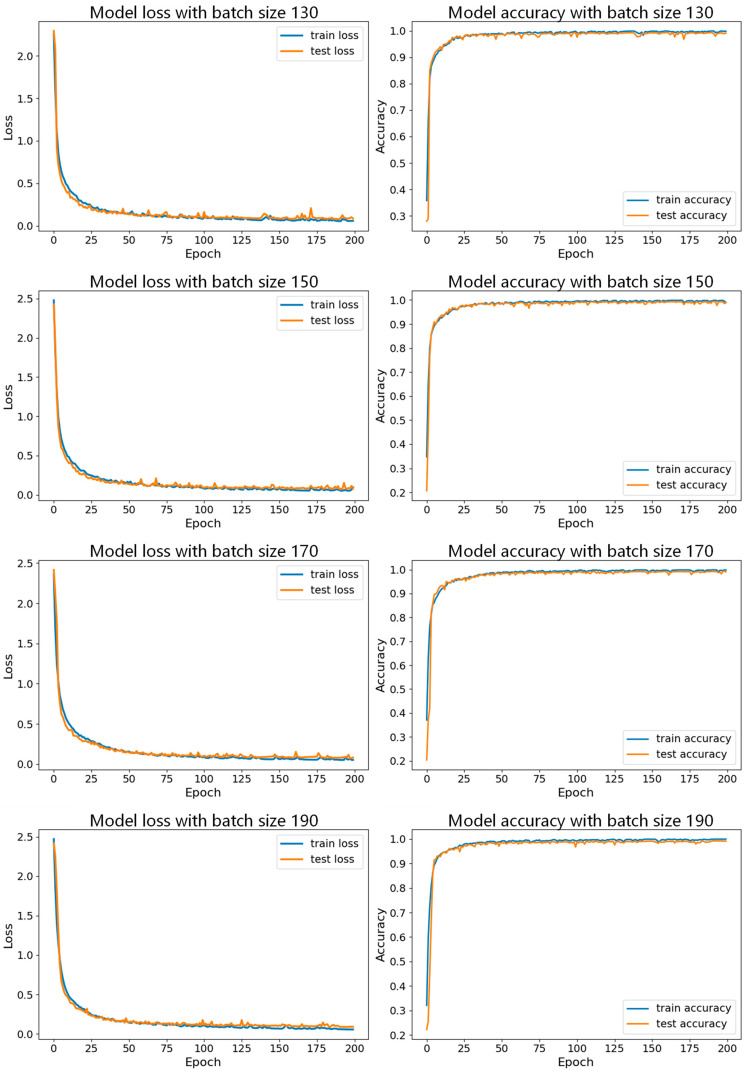
Comparison of model performance for batch sizes of 130, 150, 170, and 190 with learning rate of 0.001.

Then, with the learning rate fixed, we conducted experiments with different batch sizes, and the specific parameter sets are shown in Table 4. Figure 21 shows the accuracy curves of these seven datasets with a learning rate of 0.001, and Figure 22 and Figure 23 show the comparison of the model performance with different batch sizes.

In Figure 21, the accuracy curve with a batch size of 150 appears smoother and less volatile, indicating that the model performs more stably during training and validation, and consistently maintaining high accuracy and effectively avoiding overfitting or underfitting. Figure 22 and Figure 23 show the comparison of model performance for various batch sizes, in which the curve with a batch size of 150 drops rapidly in the early stages of training and remains at a lower loss level in the subsequent phases. Compared to other batch sizes, the loss curve with a batch size of 150 shows less fluctuation, suggesting that the model converges faster during training and can maintain a lower validation loss.

From the comparison of the experiments above, it can be concluded that when the learning rate is set to 0.001 and the batch size parameter is 150, the CNN-LSTM-SE model exhibits the best convergence performance in both the accuracy and loss curves.

**Figure 24:**
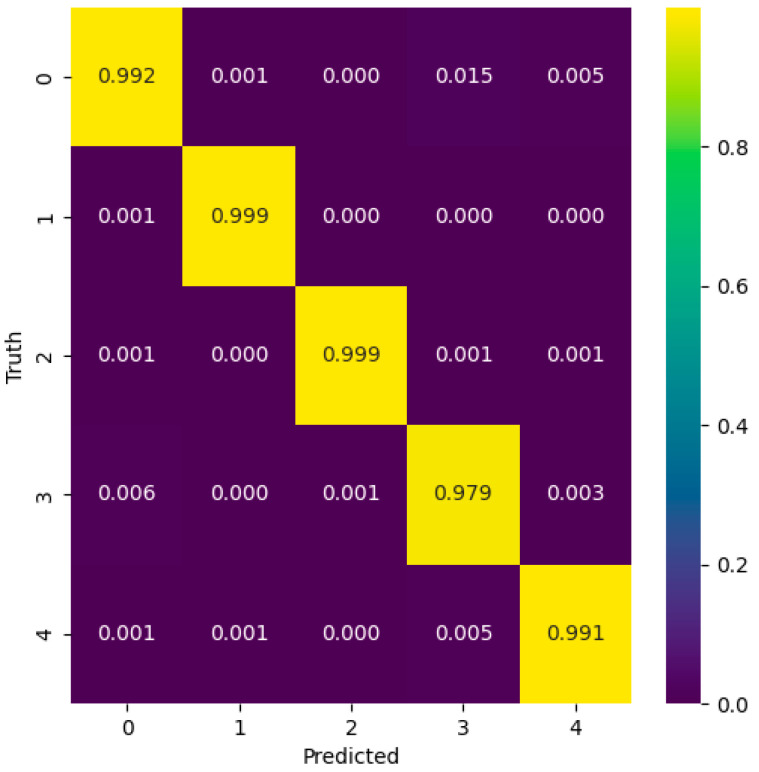
CNN-LSTM-SE model predicts confusion matrix.

This study utilized three classification evaluation indexes, Precision, Recall, and F1 Score, to assess the model’s performance. The confusion matrix of the ECG signal classification results is shown in Figure 24. The values for the three evaluation indexes of ECG-type classification are listed in Table 5 [35]. Here, the probability of classifying the ECG signals can be put into five labels: N (normal beat), A (atrial premature beat), V (ventricular premature beat), L (left bundle branch block), and R (right bundle branch block).

From the training and testing results, it is evident that the CNN-LSTM-SE classification model exhibits excellent classification performance, with all evaluation metrics showing strong results. The model achieved a prediction accuracy of 99.2% on the test set, with classification accuracy for each label reaching as high as 97%, recall rates as high as 98%, and F1 scores also as high as 98%. This largely meets the requirements for arrhythmia classification prediction. Therefore, the CNN-LSTM-SE model proposed in this paper demonstrates strong performance in ECG signal classification and recognition, with high classification accuracy.

To further validate the progress and precision of the CNN-LSTM-SE model, this paper conducted comparative experiments on the DNN, CNN, LSTM, and CNN-LSTM models using an identical dataset. Table 6 lists the performance comparison of five methods under the same dataset conditions.

It can be seen that the accuracy of the traditional DNN model is not satisfactory. The simple LSTM timing model is also not very suitable for ECG signal classification processing with high spatial features, while the CNN-LSTM model combined with the LSTM takes into account not only temporal features but also spatial features, and its accuracy is much better than that of a single feature extraction model. After adding the channel attention mechanism SE module, the accuracy of the CNN-LSTM-SE model is further improved based on the CNN-LSTM model. This shows that the CNN-LSTM-SE model has certain feasibility and superiority in the classification and prediction of arrhythmia.

## 6. Model Actual Diagnostic Testing

Although the accuracy of the model has reached a high degree, it still needs to be further tested in practical applications. This paper conducted a real human ECG signal collection and classification prediction experiment. There were 10 volunteers selected for ECG signal collection in this paper. All 10 volunteers were between 25 and 30 years old, healthy, and had no history of heart disease.

In the experiment, the subjects wore portable ECG electrode patches and used ECG signal acquisition hardware to collect ECG signals. During the collection process, the subjects kept resting, the ECG signals were collected every minute, then transmitted to the mobile terminal or PC through the hardware acquisition system, and finally saved as CSV files after denoise filtering. The trained CNN-LSTM-SE prediction model was used to make classification predictions of the target data. Table 7 lists the diagnosis result of the CNN-LSTM-SE classification model for the actual experimental ECG signal [35].

The data in Table 7 indicate that the diagnostic accuracy of normal ECG signals (N) is generally high, with most test subjects achieving an accuracy rate exceeding 90%. In certain cases, such as subject 10, the misdiagnosis rate of left bundle branch block (L) is relatively high, reaching 10.3%. This suggests that the model proposed has some errors in recognizing this type of ECG signal. The misdiagnosis rates for atrial premature contractions (A) and ventricular premature contractions (V) are fairly consistent, mostly remaining below 2%, indicating that the model proposed has a certain level of recognition ability for these types of signals.

Due to experimental limitations, the diagnostic results of the classification model have not been compared with professional ECG medical diagnostic reports. However, the physical examination reports of the subjects all indicate normal heart conditions. Overall, the CNN-LSTM-SE classification model demonstrates high overall accuracy in diagnosing actual experimental ECG signals, particularly excelling in the diagnosis of normal ECG signals. This indicates that the classification prediction model presented in this study performs well in terms of classification prediction performance, being able to accurately identify the state of the heart.

However, for certain specific types of ECG signals, such as left bundle branch block signals, the diagnostic errors suggest that the model needs further optimization or an increase in data samples to improve diagnostic performance.

## 7. Conclusions

In this paper, the classification and prediction model of arrhythmia based on the CNN-LSTM-SE fusion algorithm is implemented. This paper uses the MIT-BIH arrhythmia database as a dataset. In terms of data processing, the original data are firstly filtered through EEMD denoising, then the data are divided by R-wave positioning and ECG segmentation, the Pan–Tompkins algorithm is used for R-wave positioning, and the input data are segmented by the value of the R-wave position before and after. Oversampling and undersampling are used to deal with the problem of sample class difference. In terms of algorithm model, this paper analyzed various algorithm models, selected the fusion algorithm of CNN and LSTM, combined the advantages of CNN’s spatial feature extraction and LSTM’s temporal feature extraction, and added the channel attention mechanism to improve the performance of the model when extracting ECG signal features as much as possible. Finally, the classification of the five main types of heart conditions including normal beat, left bundle branch block, right bundle branch block, ventricular premature beat, and atrial premature beat was realized. Some performance evaluations show that the model achieves a high classification accuracy of 98.5%, with precision and recall rates exceeding 97% for all categories, which had a good performance compared with the existing commonly used ECG classification algorithms and demonstrates its potential and practical value in the field of arrhythmia diagnosis.

## Figures and Tables

**Table 1:** CNN-LSTM-SE model layer configuration and parameters.

Layer Name	Input Dimensions	Output Dimensions	Kernel Size	Pooling Size	Activation Function	Additional Information
Conv1D_1	(None, 360, 1)	(None, 120, 128)	20	/	ReLU	Batch normalization after conv.
MaxPooling_1	(None, 120, 128)	(None, 40, 128)	/	2	/	Strides = 3
Conv1D_2	(None, 40, 128)	(None, 40, 32)	7	/	ReLU	Batch normalization after conv.
MaxPooling_2	(None, 40, 32)	(None, 20, 32)	/	2	/	Strides = 2
Conv1D_3	(None, 20, 32)	(None, 20, 32)	10	/	ReLU	/
SE_Block	(None, 20, 32)	(None, 20, 32)	/	/	Sigmoid	Adaptive channel weighting
MaxPooling_3	(None, 20, 32)	(None, 10, 32)	/	2	/	Strides = 2
LSTM	(None, 10, 32)	(None, 100)	/	/	/	LSTM for temporal feature extraction
Flatten	(None, 100)	(None, 100)	/	/	/	Flattening the 3D feature map
Dropout	(None, 100)	(None, 100)	/	/	/	Dropout rate = 0.1 to prevent overfitting
Dense_1	(None, 100)	(None, 20)	/	/	ReLU	L2 regularization
Dense_2	(None, 20)	(None, 10)	/	/	ReLU	L2 regularization
Dense_3	(None, 10)	(None, 5)	/	/	Softmax	Classification output for 5 classes

**Table 2:** Noise reduction evaluation index of three methods.

Noise Reduction Method	SNR	R	RMSE
WPDN	41.3904	0.9904	1.387 × 10^−4^
VMD	40.8148	0.9916	1.5835 × 10^−4^
EEMD	62.7113	0.9999	1.0233 × 10^−6^

**Table 3:** The average accuracy and average loss rate for 7 datasets for different learning rates with a batch size of 150.

Batch Size	Learning Rate	Average Accuracy	Average Loss
150	0.0006	0.9824	0.0873
150	0.0007	0.9821	0.0910
150	0.0008	0.9828	0.0848
150	0.0009	0.9829	0.0908
150	0.001	0.9832	0.0839
150	0.0015	0.9858	0.0718
150	0.002	0.9846	0.0711

**Table 4:** Average accuracy and average loss rate for different batch sizes with learning rate of 0.001.

Learning Rate	Batch Size	Average Accuracy	Average Loss
0.001	70	0.9899	0.0540
0.001	90	0.9892	0.0596
0.001	110	0.9875	0.0640
0.001	130	0.9869	0.0669
0.001	150	0.9848	0.0744
0.001	170	0.9827	0.0799
0.001	190	0.9814	0.0883

**Table 5:** Classification evaluation index results of CNN-LSTM-SE model.

	Precision	Recall	F1-Score
N	0.992	0.981	0.986
A	0.979	0.990	0.985
V	0.991	0.993	0.992
L	0.999	0.999	0.999
R	0.999	0.998	0.999

**Table 6:** The performance comparison of five methods under the same dataset conditions.

Model Name	Accuracy Rate
DNN	93.1
LSTM	92.5
CNN	96.5
CNN-LSTM	97.1
CNN-LSTM-SE	98.5

**Table 7:** Diagnosis result of CNN-LSTM-SE classification model for actual experimental ECG signal.

	N	A	V	L	R
1	89.2%	1.9%	0.7%	6.7%	1.2%
2	91.2%	1.8%	0.7%	5%	1.1%
3	97.4%	0.3%	0.7%	1.2%	0.3%
4	99.3%	0.1%	0.2%	0.3%	0.1%
5	92.9%	1.4%	0.4%	3.9%	1.4%
6	96%	0.8%	0.2%	2.6%	0.5%
7	87.7%	1.6%	0.5%	9.2%	1%
8	94.4%	2.8%	2.1%	0.3%	0.4%
9	90.1%	1.7%	0.5%	6.2%	1.6%
10	86%	1.8%	0.6%	10.3%	1.8%

## Data Availability

The relevant data used to support the findings of this study are available from the corresponding author upon request.
